# Novel Bacteriophages Containing a Genome of Another Bacteriophage within Their Genomes

**DOI:** 10.1371/journal.pone.0040683

**Published:** 2012-07-17

**Authors:** Maud M. Swanson, Brian Reavy, Kira S. Makarova, Peter J. Cock, David W. Hopkins, Lesley Torrance, Eugene V. Koonin, Michael Taliansky

**Affiliations:** 1 The James Hutton Institute, Invergowrie, Dundee, United Kingdom; 2 National Center for Biotechnology Information, National Library of Medicine, National Institutes of Health, Bethesda, Maryland, United States of America; Cairo University, Egypt

## Abstract

A novel bacteriophage infecting *Staphylococus pasteuri* was isolated during a screen for phages in Antarctic soils. The phage named SpaA1 is morphologically similar to phages of the family *Siphoviridae*. The 42,784 bp genome of SpaA1 is a linear, double-stranded DNA molecule with 3′ protruding cohesive ends. The SpaA1 genome encompasses 63 predicted protein-coding genes which cluster within three regions of the genome, each of apparently different origin, in a mosaic pattern. In two of these regions, the gene sets resemble those in prophages of *Bacillus thuringiensis kurstaki str. T03a001* (genes involved in DNA replication/transcription, cell entry and exit) and *B. cereus* AH676 (additional regulatory and recombination genes), respectively. The third region represents an almost complete genome (except for the short terminal segments) of a distinct bacteriophage, MZTP02. Nearly the same gene module was identified in prophages of *B. thuringiensis* serovar *monterrey* BGSC 4AJ1 and *B. cereus* Rock4-2. These findings suggest that MZTP02 can be shuttled between genomes of other bacteriophages and prophages, leading to the formation of chimeric genomes. The presence of a complete phage genome in the genome of other phages apparently has not been described previously and might represent a ‘fast track’ route of virus evolution and horizontal gene transfer. Another phage (BceA1) nearly identical in sequence to SpaA1, and also including the almost complete MZTP02 genome within its own genome, was isolated from a bacterium of the *B. cereus*/*B. thuringiensis* group. Remarkably, both SpaA1 and BceA1 phages can infect *B. cereus* and *B. thuringiensis*, but only one of them, SpaA1, can infect *S. pasteuri*. This finding is best compatible with a scenario in which MZTP02 was originally contained in BceA1 infecting *Bacillus spp*, the common hosts for these two phages, followed by emergence of SpaA1 infecting *S. pasteuri*.

## Introduction

Viruses are the most abundant entities in the biosphere. In marine and soil habitats, the number of virus particles exceeds the number of cells by at least an order of magnitude [1]–[3]. Numerous viruses infect organisms from all branches of cellular life. However, virus research has traditionally focused on viruses that infect humans, other vertebrates and plants due to the obvious medical and agricultural importance of these viruses. In addition, viruses infecting several model bacteria (bacteriophages) have been studied in detail thanks primarily to their utility as tools of molecular biology. Viruses from diverse environments are incomparably less thoroughly characterized but recently environmental genomics and metagenomics of viruses have become rapidly growing research areas [4]–[7].

A total of about 2300 viruses are recognized by the International Committee on Taxonomy of Viruses [8] but this is likely to be a gross underestimate because of the enormous diversity of viruses in unsampled or poorly investigated habitats (see for example, [9], [10]. Virus particles are abundant in air, water and soils [1], [5], [11]–[15]. Recent metagenomic analyses have revealed hitherto unknown diverse assemblages of viruses in these environments [6], [9], [10], [16], [17]. For example, Fierer *et al.* [10] reported that the majority of the 4577 virus-related nucleotide sequences found in soils from different ecosystems showed no similarity to previously described sequences. Analysis of metagenomic data suggests novel patterns of virus evolution and reveals new groups of viruses providing unprecedented insights into the composition and dynamics of the virus world [7]. Viruses, in particular transducing bacteriophages, have been long known to make major contributions to gene exchange between bacteria [18]. Recently, a distinct class of defective bacteriophages, the Gene Transfer Agents (GTAs) [19], have been characterized as apparent dedicated vehicles for horizontal gene transfer that might account for extensive gene flow in bacterial and archaeal communities [19], [20]. Furthermore, viruses have emerged as a major force shaping the geochemistry and ecology of diverse environmental ecosystems [5], [21]–[23].

Tailed dsDNA bacteriophages account for 95% of all known bacterial viruses, and possibly make up the majority of phages on the planet [24]. They belong to the order *Caudovirales* which consists of three families: *Myoviridae* (long rigid contractile tails), *Siphoviridae* (long flexible non-contractile tails) and *Podoviridae* (short contractile tails) [8], [25]–[27]. One of the key features of the genomes of *Caudovirales* is their apparent mosaic architecture; in essence, each genome is a unique set of modules with different evolutionary histories that have been horizontally exchanged among phages [28]–[30].

In this work we describe a novel phage genome architecture where one phage genome nestles inside the genome of another phage, similar to a “Russian Doll” arrangement. We show that bacteriophages SpaA1 and BceA1, obtained from the bacterium *Staphylococus pasteuri* and a bacterium belonging to the *Bacillus cereus*/*B. thuringiensis* group respectively, and isolated from a soil sample from the Garwood Valley, Southern Victoria Land, Antarctica, harbor almost the complete sequence of the bacteriophage MZTP02 that had been identified previously in China [31].

## Results

### Isolation and Morphology of SpaA1

**Figure 1:**
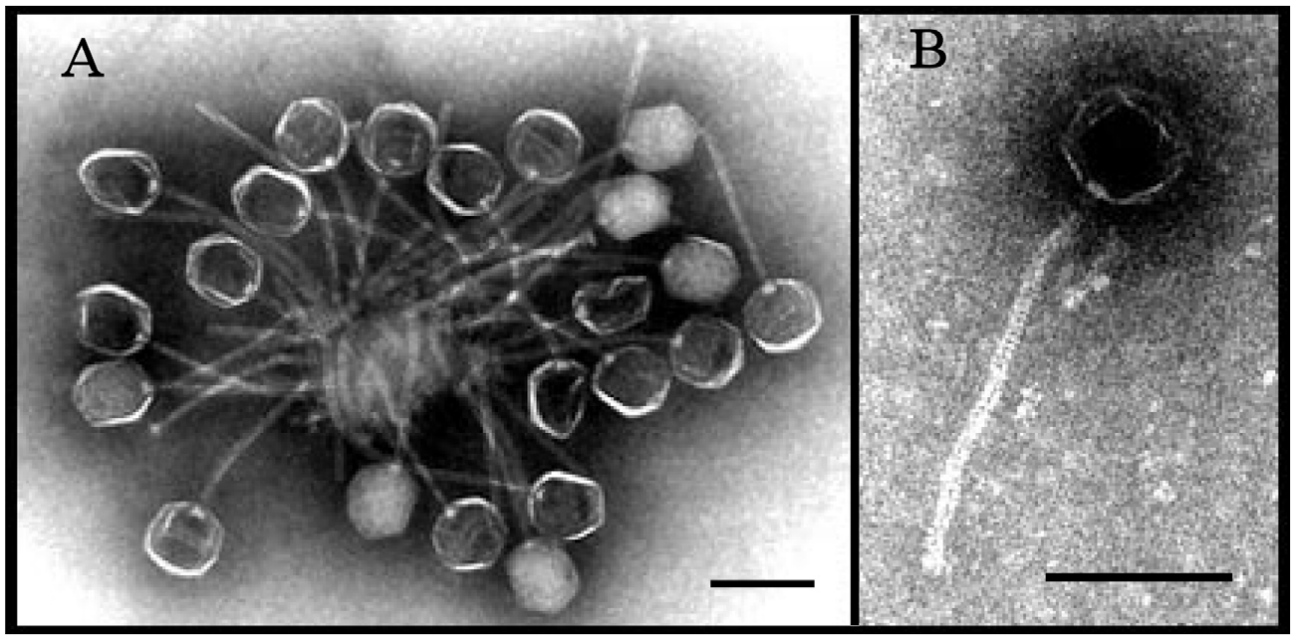
Transmission electron micrographs of phage virions showing their isometric heads and long non-contractile tails. Panel A shows multiple SpaA1 virions and panel B shows a single Bce A1 (B) virions. All scale bars represent 100 nm.

A novel temperate bacteriophage, named SpaA1, was isolated from *Staphylococus pasteuri* recovered from soils of the Garwood Valley, Southern Victoria Land, Antarctica. Bacterial cultures were grown from single colonies in liquid nutrient medium in the presence of mitomycin C to induce prophages from lysogenic bacteria. SpaA1 was isolated from the growth medium and examined by transmission electron microscopy (TEM) (Figure 1A). The morphology of SpaA1 is typical of the *Siphoviridae* family of phages. SpaA1 virions have isometric heads (B1 morphotype) with a diameter of ∼63 nm. The virion tails are ∼210 nm long and appear to be flexible and non-contractile.

### General Features of the SpaA1 Genome

**Figure 2:**
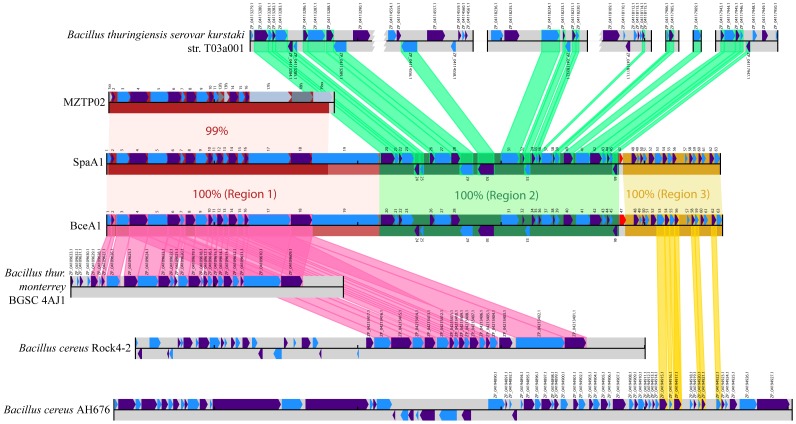
Architectures of SpaA1, BceA1 and MZTP02 genomes: comparison with BLAST protein matches to phage proteins in four *Bacillus* genomes. The horizontal bars represent DNA sequences (all to scale) with annotated CDS on the forward (upper) or reverse (lower) strand shown as pointed boxes, generally in alternating blue and purple. The red, green and yellow shading indicates the three functional modules of phages SpaA1 and BceA1 (center) which are 100% identical except for the area around ORF47 (bright red), and the 99% nucleotide identical matching region in module I with phage MZTP02 (second row from top). Rather than the original annotation for MZTP02, annotation based on SpaA1/BceA1 genome analysis (Table 1) is shown, with grey colouring for partial sequences (1 and 19), and genes with frame shifts (12, 13, 17, 18). The bottom three bars represent complete contigs from three separate *Bacillus* genomes, with red/yellow highlighting top BLAST matches from SpaA1/BceA1 module I and III proteins, showing synteny visually. The top row of bars represents seven contigs from another *Bacillus* draft genome with green highlighting for BLAST protein matches from SpaA1/BceA1 module II proteins. Three of these contigs have been truncated for display. For clarity, additional BLAST matches to other contigs from these bacterial genomes are not shown (e.g. SpaA1/BceA1 ORF37 matches another contig in *B. thuringiensis* var. *monterrey* BGSC A4J1). This figure was drawn using GenomeDiagram [61] and Biopython [62].

The genome of phage SpaA1 consists of 42,784 bp flanked by complementary 9-bp single stranded cohesive (*cos*) ends (5′-…TGGAGGAGG -3′ and 3′-CCTCCTCCA…-5′). Using GeneMark.hmm [32], 63 open reading frames (ORFs) were identified as probable protein-coding genes. The predicted proteins encoded by these 63 ORFs were compared to the non-redundant protein sequence database (National Center for Biotechnology Information, NIH, Bethesda) using PSI-BLAST [33] and the Conserved Domain Database using RPS-BLAST [34]. Analysis of the most similar proteins (best hits) for all predicted gene products of SpaA1 reveals three major regions of apparent different origins suggesting a modular architecture of the genome (Figure 2; Table 1).

**Table 1:** Open Reading Frames in the genomes of SpaA1 and BceA1.

					Best matches to the closely related prophages					
ORF	Strand	Start	Stop	Amino Acids	*B. thuringiensis israelensis* ATCC 35646	*B. thuringiensis kurstak* str. T03a001	*B. thuringiensis monterrey* BGSC 4AJ1	*B. cereus* AH676	Comments on poorly characterized proteins	Annotation
**Structural Module (MZTP02-related)**										
1	+	8	337	107		ZP_04117829.1	ZP_04109629.1		Present in many prophages and phages only of Bacillus species	Conserved phage protein
2	+	324	536	68	ZP_00742394.1	ZP_04117831.1	ZP_04109627.1		Present in many prophages and phages only of *Bacillus* species	Conserved phage protein
3	+	687	1601	302			ZP_04109626.1			Phage terminase, small subunit
4	+	1591	2865	422			ZP_04109625.1			Phage terminase, large subunit
5	+	2878	4311	475			ZP_04109624.1			Phage portal protein, SPP1
6	+	4235	5152	303			ZP_04109623.1			Phage minor head protein
7	+	5152	5424	88			ZP_04109622.1		Homolog of gp34.65 of *Bacillus* phage SPO1, DUF2829 family protein, present in a wide range of bacteria, phages and prophages	Conserved phage protein, gp64 family
8	+	5504	6184	224			ZP_04109620.1		Belongs to DUF4355 family; present in a wide range of bacteria, phages and prophages	Phage scaffold protein
9	+	6202	7044	278			ZP_04109619.1		Capsid protein of gp6 family (gp6 proteins of Mycobacterial phages, eg. Mycobacterium phage Che8)	Phage capsid protein gp6
10	+	7095	7403	100			ZP_04109617.1		Head-tail connector protein, homolog of gp15 of Bacteriophage SPP1	Head-tail connector protein, gp15 family
11	+	7400	7744	112			ZP_04109616.1			Phage head-tail adaptor protein
12	+	7719	8126	133			ZP_04109615.1			Phage portal protein, HK97 family
13	+	8132	8494	118			ZP_04109614.1		Present mostly in prophages and phages of Firmicutes; homolog of gp11 of *Bacillus* phage TP21-L	Phage structural protein
14	+	8509	9102	195			ZP_04109613.1		Present in a wide range of phages and prophages	Conserved phage protein
15	+	9149	9577	140			ZP_04109612.1		Present in many phages and prophages, mostly of Firmicutes	Conserved phage protein
16	+	9613	9888	89			ZP_04109611.1		Present in prophage regions of several *Bacillus* species	Conserved phage protein
17	+	9889	12828	977			ZP_04109610.1			Phage tail tape measure
18	+	12841	14319	490		ZP_04117974.1	ZP_04109609.1			Phage tail fiber protein
19	+	14316	19025	1567	ZP_00741743.1	ZP_04117933.1	ZP_04112413.1			Phage minor structural protein
Replication module										
20	+	19125	20084	319		ZP_04113280.1				Phage integrase
21	+	20098	20379	93		ZP_04113281.1			Conserved in a wide range of bacteria, phages and prophages; DUF4055 family protein; has a coiled-coil domain	Uncharacterized secreted or membrane protein
22	+	20382	20594	70		ZP_04113282.1				Holin, phage phi LC3 holin homolog
23	+	20594	21412	272		ZP_04113283.1	ZP_04108738.1			N-acetylmuramoyl-L-alanine amidase
24	-	21453	21782	109		ZP_04113284.1			Conserved in a wide range of bacteria, phages and prophages; DUF2614 family	Membrane protein, contains Zn-finger
25	-	21851	22072	73		ZP_04113285.1				XRE family transcriptional regulator
26	+	22535	22855	106		ZP_04113286.1	ZP_04108441.1		Present in prophages and a few other genomic regions of several Bacillus species	Phage membrane protein
27	+	22866	24032	388		ZP_04113287.1	ZP_04108442.1			Cell division protein FtsK/SpoIIIE
28	+	24022	24630	202		ZP_04113288.1	ZP_04108443.1			DNA-binding protein containing HTH domain
29	-	24635	25516	293	ZP_00743542.1	ZP_04113289.1				DNA replication protein, HTH and DnaB-like domains
30	-	25890	26990	366	ZP_00741791.1	ZP_04114556.1				DNA integration/recombination/inversion protein
31	+	27508	28746	412		ZP_04118234.1				DNA-binding protein containing XRE family HTH domain
32	+	28987	29118	43		ZP_04118233.1			Present in several prophage regions of Bacillus species	Conserved phage protein
33	-	29146	29490	114		ZP_04118232.1	ZP_04111960.1			XRE family transcriptional regulator
34	+	29639	29875	78		ZP_04118231.1	ZP_04111959.1			XRE family transcriptional regulator
35	+	29908	30096	62		ZP_04118230.1				Transcriptional regulator, pbsX
36	+	30121	30276	63		ZP_04118115.1	ZP_04123682.1		Conserved in a wide range of bacteria, phages and prophages of Firmicutes	Conserved phage protein
37	+	30322	31101	64			ZP_04111906.1	ZP_04195192.1		Antirepressor
38	+	31126	31242	65		ZP_04117906.1			Found in several prophage regions of *Bacillus* species	Phage membrane or secreted protein
39	+	31263	31577	112		ZP_04117905.1			Conserved in a wide range of phages (including several unclassified dsDNA phages) and prophages of Firmicutes	Conserved phage protein
40	+	31852	32499	215		ZP_04117909.1				Sigma70, RNA polymerase sigma factor
41	+	32723	33739	338		ZP_04117943.1	ZP_04112661.1			DnaD-like replication protein
42	+	33702	34514	270		ZP_04117944.1	ZP_04112587.1			Predicted DNAreplication ATPase related to DnaC
43	+	34556	34822	88		ZP_04117945.1			Present in several prophage regions of Bacillus species and found in Bacillus phage IEBH	Conserved phage protein
44	+	34894	35058	54		ZP_04117946.1			Conserved in a wide range of bacteria, phages and prophages of Firmicutes; DUF3954 family	Conserved phage protein
45	+	35076	35291	71	ZP_00742082.1					XRE family transcriptional regulator
46	-	35288	35587	99	ZP_00742083.1	ZP_04117947.1				wHTH containing DNA binding protein, YjcQ family
**Additional Regulatory and Recombination Module**										
47 SpaA1	+	35737	35991	84		ZP_04117960.1			Present in prophage regions of a few *Bacillus* species, distantly related to Gp40 protein of *Clostridium* phage phi3626	Conserved phage protein
47 BceA1		35738	36208	156		ZP_04112932.1			Present in prophage regions of a few *Bacillus* species	Conserved phage protein
48	+	36597	36959	120		ZP_04117741.1			Present in phages prophage regions of several Bacillus species	Conserved phage protein
49	+	36993	37169	58			ZP_04109637.1		Found in phages and prophage regions of Firmicutes, several homologs in *Streptococcus* and *Staphylococcus*, including PH10_gp22 of Streptococcus phage PH10	Conserved phage protein
50	+	37205	37402	65					No detectable homologs	Hypothetical protein
51	+	37399	37677	92					Conserved in a wide range of bacteria, phages and prophages of Firmicutes;	Conserved phage protein
52	+	37797	38183	128					Conserved in a wide range of bacteria, phages and prophages mostly of gram-positive organisms; Structure available (PDB:2OX7)	Conserved phage protein, YopX superfamily
53	+	38214	38744	176				ZP_04194915.1		Recombination protein U
54	+	38764	39081	105				ZP_04194916.1	Present in a wide range of prophage regions of Firmicutes species; often as separately encoded Zn-finger and HTH domains	Zn-finger and HTH domain containing protein
55	+	39108	39446	110				ZP_04194917.1		Sigma70, RNA polymerase sigma factor, positive control factor Xpf
56	+	39446	39730	94				ZP_04194917.1		Sigma70, RNA polymerase sigma factor, positive control factor Xpf
57	+	40317	40493	58					No detectable homologs	Hypothetical protein
58	+	40623	40865	80				ZP_04194920.1	One homolog present in the prophage region of *Bacillus cereus* AH676	Hypothetical protein
59	+	40858	41124	88		ZP_04117814.1		ZP_04194921.1	Found in many prophages and phages only of Bacillus species	Conserved phage protein
60	+	41263	41505	80	ZP_00741808.1				Homolog of gp34.65 of *Bacillus* phage SPO1, DUF2829 family protein, found in a wide range of bacteria, phages and prophages	Conserved phage protein
61	+	41505	41768	87	ZP_00741808.1				Homolog of gp34.65 of *Bacillus* phage SPO1, DUF2829 family protein, found in a wide range of bacteria, phages and prophages	Conserved phage protein
62	+	42028	42348	106			ZP_04112467.1	ZP_04194922.1	Present in prophage regions of several *Bacillus* species	Conserved phage protein
63	+	42376	42759	127			|		No detectable homologs	Hypothetical protein

The nucleotide start and stop codon positions for the SpaA1 ORFs are indicated; for the alternative ORF47, gene coordinates of BceA1 also are provided. Best matches are shown for four *Bacillus* genomes that contain most similar prophage regions. DUF, Domain of Unknown Function family in the PFAM database.

The nucleotide sequence of the first module (left and coloured red in Figure 2) of the SpaA1 genome is almost identical to the sequence of the entire 15,717 bp genome of another bacteriophage, MZTP02 (apart from its 5′ - and 3′- terminal regions of 41 bp and ∼370 bp long, respectively) that was isolated from *Bacillus thuringiensis*, strain MZ1 in China [31] (Figure 2). Unlike SpaA1 DNA which contains terminal *cos* ends, MZTP02 DNA contains 40-bp terminal inverted repeats and its 5′-terminus is covalently bound to a terminal protein presumably encoded by ORF9 (according to our annotation; [31]). Interestingly, an almost identical sequence is present as a prophage in the genome of *B. thuringiensis* var. *monterrey* BGSC 4AJ1 (locus IDs: bthur0007_34460 to bthur0007_34660, accession no. NZ_CM000752.1) and *B. cereus* Rock4-2 (locus IDs: bcere0023_35280 to bcere0023_35430, accession no. NZ_ACMM01000283.1). The 19 potential ORFs located in this region encode predicted structural proteins and proteins involved in assembly of SpaA1 and thus form the “structural” module of the genome. The architecture of this module in SpaA1 shows features that are typical of other bacteriophages of the family *Siphoviridae*. In particular, there is clear synteny among genes encoding virion subunits and proteins involved in virion assembly [29]. The genes for head and tail assembly are encoded in the same transcriptional orientation, with the head genes located upstream of the tail genes (Figure 2 and Table 1). The predicted head genes include the large and small terminase subunits (ORF3 and ORF4, respectively), the portal protein (ORF5), the minor capsid subunit (ORF6), the scaffold protein (ORF8), gp-like tail connector (ORF1) and head-tail adapter (ORF11); the tail genes include the major tail subunit (ORF12) and the tape measure protein (ORF17), followed by the tail fiber protein (ORF18) and the minor tail protein (ORF19) (Table 1). The length of the tape measure protein gene corresponds to the length of the phage tail and is thus commonly the largest gene in the genome [29]. In SpaA1, however, the tape measure protein (979 aa) is only the second largest protein, the largest being the minor tail structural protein (1569 aa). Bacillus phage TP21-L also has a minor structural protein that is larger than the tape measure protein [35]. For most of the known phages, the size of the tape measure protein corresponds to a fairly constant 0.15 nm of tail length per amino acid residue [36]. However, the tail length-to-amino acid ratio for SpaA1 is ∼0.20 nm per amino acid residue, suggesting that this protein might be somewhat more extended than those in other known phages.

The gene arrangement in the second SpaA1 genome module (coloured green in Figure 2), which consists of genes with functions in DNA integration, replication, transcription, cell entry and exit (ORF20–ORF46), and may be denoted the ‘replication module’, is very similar to the organization of the corresponding regions in several prophages of *B. thuringiensis* Kurstaki strain (Figure 2, Table 1). The longest conserved gene array (locus_ID: bthur0006_5910 to bthur0006_6000; accession no. NZ_CM000751.1) contains the first 10 ORFs in this region. In particular, the replication module encompasses five predicted transcriptional regulators (ORFs 25, 33–35 and 45) and four putative DNA-binding proteins (ORFs 24, 28, 31, and 46). Other ORFs related to replication in this module include ones encoding a FtsK/SpoIIIE- like protein (ORF27), and three proteins containing HTH and DnaB domains (ORF29), a DnaD domain (ORF41) and a predicted ATPase related to DnaC (ORF42). The module also encodes an antirepressor (ORF37), two proteins involved in cell lysis (ORFs 22 and 23) and two integrases, ORF20 which shows 95% amino acid sequence identity with the integrase of prophage lamdaBa02 (accession number EEM54966.1), and ORF30 which shows 80% amino acid sequence identity with an integrase from *B. thuringiensis* (accession number EAO53934.1).

The third genomic module (coloured yellow in Figure 2) of SpaA1 is similar to a portion of *B.cereus* AH676 prophage and contains additional regulatory and recombination related genes including a potential recombination protein U (ORF53) and a potential DNA-binding protein (ORF54). ORFs 55 and 56 are similar to the N-terminal and C-terminal parts of an RNA polymerase sigma 70 factor, respectively. The last nucleotide of the TAA termination codon of ORF55 is also the first nucleotide of the ATG initiation codon of ORF56 within a TAATG sequence. However, the reading frame of ORF56 extends 5′ without an initiation codon to nucleotide 39374 in SpaA1, and a -1 frameshift in the region of nucleotides 39385–39390 during translation of ORF55 could result in a single protein of 206 amino acids which is similar to an intact RNA polymerase sigma factor from *B. cereus* (accession number ACM16007.1). Interestingly, approximately 70% of dsDNA long-tailed phages including siphoviruses exploit the programmed frameshift mechanism for gene expression and the majority of frameshift candidates appear to use a -1 frameshift [37]. However, no canonical -1 frameshift signal has been detected by KnotInFrame, a tool for the prediction of ribosomal frameshift events [38]. Alternatively, ORF55 and ORF56 might encode two distinct proteins possibly forming a two-subunit complex. ORF40 of SpaA1 encodes a second RNA polymerase sigma 70 factor that is not closely related to the ORF55/56 sigma factor and is most similar to a homolog from *B. thuringiensis* (accession number EEM99580.1). The longest region of synteny conservation between SpaA1 and AH676 contains 6 ORFs (locus_ID: bcere0027_53380 to bcere0027_53450; accession no. NZ_CM000738.1).

**Figure 3:**
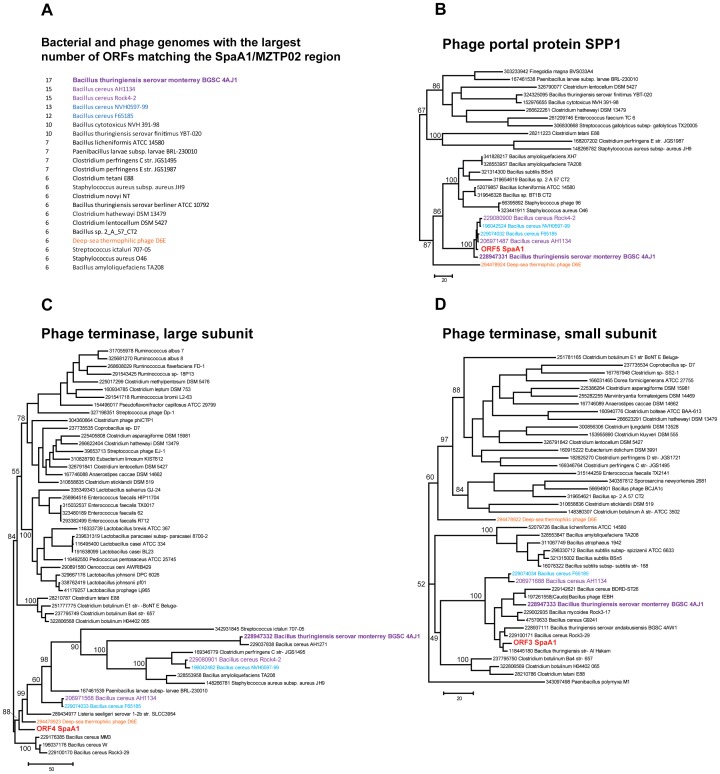
Phylogenetic analysis of selected SpaA1 genes. A. Bacterial and phage genomes sorted by the number of ORFs matching the SpaA1/MZTP02 region (based on the up to 200 best hits in NR database). On the left, the actual number of hits is indicated. Color code: three bacterial genomes with the 17-15 ORFs matching the SpaA1/MZTP02 region:purple; three bacterial genomes with the 13-12 matching ORFs: light blue; the phage with the largest number of hits matching the SpaA1/MZTP02 region:orange. B, C, D. Unrooted maximum likelihood trees for three ORFs the SpaA1/MZTP02 region. Each terminal tree node is labelled with GenBank Identifier (GI) number and full systematic name of an organism. Color code is the same as in the Figure 3A. The SpaA1 phage sequences are shown in red. Bootstrap support (percentage) are indicated for selected internal branches.

Phage terminase genes can be used to construct phylogenetic trees which correlate with the structure of the phage DNA termini [39]. However, we have detected evidence of recombination in the MZTP02 region that encompasses at least the gene for the large terminase subunit of SpaA1. The majority of the ORFs within the ORF1-ORF18 region (the MZTP02sequence) show best hits into several Bacilli genomes (Figure 3A), and the tree for phage portal protein SPP1, taken as a typical example, clearly demonstrates clustering with sequences from these organisms (Figure 3B). In contrast, the tree for ORF4, the large subunit of phage terminase, shows very different topology (Figure 3C), suggesting that notwithstanding the synteny in this region (Figure 2), ORF4 appears to have been acquired from a different, unknown source. The topology of the tree for ORF3, the small subunit of phage terminase, was compatible with the typical, SPP1-like topology (Figure 3B and 3D). Thus, the large subunit gene apparently was displaced *via* ‘in situ’ recombination [40], an observation that further emphasizes the mosaicism in the phage genomes.

Neither the second nor the third genomic modules of SpaA1 completely match any known prophages or phages. Even with the most closely related phages, such as Cherry [41], EJ [42], phBC6A51 [43] and the deep-sea thermophilic phage D6E, [44] there are only a few significantly similar predicted proteins (Figure 3A and Table 1) indicating that SpaA1 represents a novel group of tailed phages.

The overall G + C content of the phage is 35.63% strongly resembling its host *S. pasteuri* (35%, [45]) as well as the host for MZTP02 (*B. thuringiensis*, 35.3%, [46]). No significant differences in the GC content were detected among the three genomic modules of SpaA1.

### The BceA1 Bacteriophage

A further search and characterization of bacteriophages from Antarctic soils identified another temperate bacteriophage, named BceA1, from a bacterium of the *B. cereus*/*B. thuringiensis* group. The morphology of BceA1 is very similar to that of SpaA1 and hence is typical of the *Siphoviridae* family. BceA1 virions also had isometric heads with a diameter of ∼63 nm and flexible tails of ∼210 nm in length (Figure 1B). The genome of phage BceA1 consists of 42,932 bp and like SpaA1 encompasses 63 ORFs. These two phages are identical apart from ORF47 and the immediate surrounding area; the SpaA1 ORF47 encodes a protein of 84 aa and BceA1 a protein of 156 aa. These two proteins have non-overlapping sets of homologs and hence appear to be unrelated (Figure 2, Table 1 and data not shown). Although the functions of both these proteins are unknown, it seems plausible that they are directly involved in the control of the host range as both SpaA1 and BceA1 could infect *B. cereus* but only SpaA1 could infect *S. pasteuri* (Table 2).

**Table 2:** SpaA1 and BceA1 host specificities on *S. pasteuri* and *B*. *cereus.*

Bacterium	SpaA1 titer^a^	BceA1 titer^a^
*S. pasteuri*	1.4×10^8^	1.7×10^7^
*B. cereus*	<10	5.0×10^7^

a Titers are expressed in PFU per milliliter. Means were determined on the basis of the results of three different infections.

### Host Ranges of SpaA1 and BceA1

SpaA1 and BceA1 inocula were used to infect *B. cereus* and *S. pasteuri* in a plaque assay. BceA1 produced plaques with a titre of greater than 10^7^ plaque forming units (pfu)/ml on both bacterial species but SpaA1 produced plaques with a high titre only on *S. pasteuri* (Table 2).

## Discussion

### The Entire MZTP02 Genome is a Potentially Independent Mobile Element

As pointed out above, the nucleotide sequence of the “structural” module of the SpaA1 and BceA1 genomes is 99% identical to the sequence of the entire genome of another bacteriophage, MZTP02 (apart from short 5′ - and 3′- terminal regions) [31]; (Figure 2). SpaA1 and BceA1 are similar in this respect to phage N15 which acquired a module encoding head and tail protein genes from a lambda-like phage [47]. However SpaA1 is the first finding of an almost complete phage genome within the genome of another phage. The presence of similar inserts in the genomes of *B. thuringiensis* var. *monterrey* BGSC 4AJ1 and *B. cereus* Rock4-2 in the form of a prophage (Figure 2) suggests that the (nearly) complete MZTP02 genome can travel between genomes as a distinct entity. The MZTP02 genome does not contain any identifiable integrase genes so a question arises as to how it became integrated into these genomes. It is possible that MZTP02 does not integrate on its own but rather exists as a linear prophage in the same way as GIL01 [48]. The MZTP02 and GIL01 genomes are both ∼15 kbp long and contain inverted terminal repeats and 5′ terminal genome-linked proteins [31], [48]. MZTP02 could then possibly recombine with a separate co-infecting phage and this could have led to the integration of the resulting composite phage genomes into the bacterial chromosome. Alternatively, the integrase of a co-infecting phage could facilitate integration of MZTP02. The MZTP02 genome encodes only virion subunits as well as proteins involved in DNA packaging and capsid assembly (Table 1). We hypothesise that MZTP02 is likely to be a satellite virus as it does not encode proteins required for DNA replication and transcription, and more importantly, proteins involved in cell entry and exit. If this is the case then MZTP02 probably is unable to infect and replicate in a host bacterium by itself, but rather depends on co-infection of the host with a helper virus that remains to be identified. MZTP02 infected six different *B. thuringiensis* strains [31] suggesting that such a putative helper phage must be fairly ubiquitous among *B. thuringiensis* strains, possibly as an integrated prophage. A thoroughly studied satellite bacteriophage is P4, also regarded as a natural phasmid (phage-plasmid), which depends on phage P2 for reproduction in *Escherichia coli* [49]. However, in contrast to MZTP02, P4 possesses genes essential for DNA replication, but depends on P2 helper genes for the head and tail morphogenesis and for lysis of the host cell [50]. The size of the head of SpaA1 is ∼63 nm which contrasts with the size of 84 nm reported for MZTP02 [31]. In the P2/P4 helper virus system, two different capsid sizes are produced from proteins encoded by P2 and a size-determining protein encoded by P4 produces smaller capsids to package the smaller P4 DNA [51]. SpaA1 might encode an unidentified size-determining protein that produces smaller capsids. A capsid of ∼84 nm in size might seem large to encapsidate the 15.7 kb genome of MZTP02 but it is conceivable that multiple copies of its genome are encapsidated in such capsids in a similar way in which three copies of the P4 genome can be encapsidated in P2 size heads [52].

### Evolutionary Relationships between SpaA1/BceA1 and MZTP02

The 99% sequence identity over 15 kbp in the SpaA1/BceA1 and MZTP02 genomes obviously points to an evolutionary link between these bacteriophages. However, the precise nature of this link remains unclear given that, firstly, these phages were isolated from geographically distant regions; SpaA1 and BceA1 in Antarctica and MZTP02 in China, and secondly, SpaA1 and MZTP02 were isolated from different host species; *Staphylococcus* and *Bacillus*, respectively. The presence of a sequence identical to the nearly complete genome of MZTP02 in the genome of SpaA1 suggests the existence of a common host and a common habitat for the two viruses in the recent past. It seems likely that this common host is a bacterium of the genus *Bacillus*. Indeed, BceA1 which is nearly identical in sequence to SpaA1 and also includes the almost complete MZTP02 genome within its own genome, was isolated from a bacterium of the *B. cereus*/*B. thuringiensis* group. The discovery of identical phage sequences in habitats as geographically and ecologically distant as Antarctica and China might seem puzzling. However, numerous studies have reported global distribution of at least some bacteriophages [9], [53] and the present results suggest that MZTP02 belongs to this class of ubiquitous phages. There are two alternative evolutionary scenarios to account for the relationship between MZTP02 and SpaA1. Firstly, an ancestor of SpaA1 might have possessed a structural module homologous to MZTP02, and MZTP02 arose as a result of excision from the ancestral SpaA1/BceA1-like phage. Alternatively MZTP02 might have evolved elsewhere with subsequent recombination leading to the integration of MZTP02 into the genome of an ancestor of BceA1/SpaA1 and replacement of the original structural module of that ancestral phage with the structural module of MZTP02. Our experiments showed that both SpaA1 and BceA1 phages can infect *B. cereus*, but only one of them, SpaA1, is able to infect *S. Pasteuri*. These findings are best compatible with a scenario in which MZTP02 and BceA1 first evolved in *Bacillus spp*, the common hosts for these two phages, whereas SpaA1 evolved later, after ORF47 was replaced in BceA1 by an unrelated gene.

The findings reported here indicate that MZTP02 is not only a satellite phage but also an independent mobile module that occurs in the genomes of phages and prophages, resulting in chimeric viral genomes. To our knowledge, such nested architecture of a phage genome has not been described previously and seems to indicate that complete viral genomes could play an even greater role in genetic exchanges in the prokaryote world than previously suspected.

## Materials and Methods

### Ethics Statement

All necessary permits were obtained for the described field studies. The Garwood Valley falls within the McMurdo dry valleys Antarctic specially managed area (ASMA) no. 2 designated under the Protocol on Environmental Protection to the International Antarctic Treaty. Entry to and field operations in the ASMA (including sampling and removal of soils, rocks, organisms and water) for the research described here is regulated by a permit issued to field party K052, which included D.W. Hopkins, by Antarctica New Zealand, The International Antarctic Centre, Orchard Road, Christchurch, New Zealand.

### Isolation of Bacteria from Antarctic Soil

A soil sample was collected in the Garwood Valley, Antarctica (78′01°S, 163′53°E; Ross Dependency Ross Sea region; [54]) in January 2006, at the site of a soil ecological experiment [55]. The samples were transported to the UK frozen and stored at 4°C. 1 g of soil was mixed with 100 ml sterile 0.01× nutrient broth (10^−2^ dilution) and stirred at room temperature for 1 h. Serial dilutions to 10^−5^ were made in 0.01× nutrient broth and 200 µl of each dilution was plated onto LB Agar plates and incubated at 20°C. Bacterial colonies of different appearance were chosen and sub-cultured three times on LB Agar plates.

### Induction and Isolation of Bacteriophages

A single colony of the bacterium was grown up overnight in 10 ml LB in a shaking incubator at 28°C. Cells were then centrifuged for five minutes at 3,000× g; the cell pellet was drained and resuspended in 2.5 ml 0.01 M Mg_2_SO_4,_ and 20 µl of mitomycin c (20 µg/ml) added. Cell suspensions were then shaken at 28°C for 1 h and washed twice with 2.5 ml 0.01 M Mg_2_SO_4._ Cells were finally resuspended in 10 ml LB and shaken at 28°C overnight. Bacteria were centrifuged as before and the supernatant was filtered through 0.45 µm syringe filters (Millipore Corporation, Billerica, MA 01821). Filtrate was centrifuged through a CsCl step gradient containing 1 ml of each of 1.3 g/ml, 1.5 g/ml and 1.7 g/ml CsCl in an SW41 rotor at 83,000× g for two hours at 10°C in an Optima™ L-80 XP ultracentrifuge (Beckman Coulter Inc.). The middle density layer was collected, diluted at least 1∶5 in SM medium (0.05 M Tris-HCl pH 7.5, 0.1 M NaCl, 0,01 M MgSO_4_.7H2O) and centrifuged in an R90 Ti rotor for 1.5 hours at 214,000× g. Pelleted bacteriophage particles were resuspended in a small volume of SM medium.

### Transmission Electron Microscopy (TEM)

TEM analysis of virus particles was done as follows: carbon-coated copper grids were floated for five minutes on 10 µl drops of samples on wax slides. Grids were then removed from the drops and excess sample was drained from the grids using filter paper. Then 10 µl drops of 1% (w/v) phosphotungstic acid pH 6.0–7.0 were put on the grids, left for 30 seconds and then drained from the grids using filter paper. Grids were examined in a Jeol 100 S Electron Microscope at 80 kV. Measurements of virus particles dimensions were done using Adobe Photoshop CS2.

### Identification of Bacterial Species

Bacterial hosts of isolated bacteriophages were identified by amplifying their 16 *S* ribosomal RNA genes by PCR and comparing these sequences to the GenBank database using the BLAST program available at the National Center for Biotechnology Information (http://www.ncbi.nlm.nih.gov). A single colony from a plate was mixed with 50 µl dH_2_O and heated at 95°C for 4 minutes and 2 µl was then used for PCR. PCR was carried out using Phusion DNA polymerase (Finnzymes) and primers 63F (CAGGCCTAACACATGCAAGTC) and 1387R (GGGCGGTGTGTACAAGGC). The PCR products were cut out from 1% agarose gels and purified using QIAquick gel extraction kit (Qiagen) and sequenced by Sanger capillary method using primers 63F, 1387R, V2F (GAGTGGCGGACGGGTGAGTAAT), V3R (CGTATTACCGCGGCTG), V6F (TCGATGCAACGCGAAGAA) and V7R (ACATTTCACAACACGAGCTGACGA). The bacterial host of SpeA1 was identified as *Staphylococcus pasteuri* with which it had a greater than 99% identity. The bacterial host of BceA1 was identified as a member of the *Bacillus cereus/Bacillus thuringiensis* group which share greater than 99% identity in the 16 *S* ribosomal RNA gene.

### Virus Host Range Determination

The SpaA1 and BceA1 phages were propagated in LB broth on *S. pasteuri* and *B*. *cereus,* respectively. Phage preparations were added to an equivalent volume of mid-log-phase bacteria and incubated at 30°C with agitation for 24 h. Phage supernatants were recovered, and this process was repeated until a sufficiently high-titer phage stock was obtained (>10^9^/ml). All phage preparations were filter sterilized prior to use. 0.1-ml aliquots of an overnight LB broth culture were added separately to 0.1 ml of undiluted phage and each of three 100-fold serial dilutions, in four sterile, 10-ml, round-bottom polypropylene tubes. After incubation at 37°C for 15 min, 3 ml of soft LB agar was added to each tube, gently mixed by inversion, and poured over the surface of a pre-warmed LB agar plate. Plates were incubated for 24 h at 30°C, and plaques were enumerated to determine the number of PFU per milliliter.

### Isolation of Nucleic Acid from Bacteriophage Particles

Suspensions of bacteriophage particles were treated with DNase (Promega) and RNase (Promega) and incubated at 37°C for 30 minutes. The reaction was stopped by adding Stop buffer (10% (v/v) 0.02 M EGTA) and incubating at 65°C for 10 minutes. The samples were then incubated with 1/10^th^ volume of 2 M Tris-HCl pH8.5, 0.2 M EDTA, 1/20^th^ volume 0.5 M EDTA pH8 and an equal volume of formamide at room temperature for 30 minutes. Two volumes of 100% ethanol were then added and the samples kept at −20°C overnight. Samples were then centrifuged at 13,000× g 8°C in a bench-top Eppendorf 5415R for 20 minutes and the pellets washed with 70% ethanol, air-dried and resuspended in TE buffer (0.01 M Tris-HCl pH8, 0.001 M EDTA).

### 454 Sequencing of Nucleic Acids

Roche 454 sequencing was performed by GenePool (University of Edinburgh) using 2/16 of a PicoTiterPlate for each phage. For SpaA1 the FLX platform was used and gave 29338 reads with median read length 247 bp and an approximate coverage of 106× The later sample for BceA1 used the “Titanium” upgrade and gave 51597 reads with median read length 320 bp and an approximate coverage of 18×; however this was variable with regions that had no coverage and gaps were filled in by Sanger capillary sequencing (see below).

### Assembly of 454 Sequence

The 454 reads for SpaA1 were initially assembled with Roche “Newbler” gsAssembler v1.1, later v2.0, however this required manual intervention to cope with the high coverage. SpaA1 was then assembled with MIRA v3.2 [56], additional Sanger capillary sequencing done, and a hybrid assembly performed with MIRA. This gave one large contig whose ends repeated, giving a circularised sequence of approximately 43 kb, with no marked coverage variation to suggest possible end points of the phage’s linear form (visualized using Tablet, [57]). For BceA1, despite having more 454 data, de novo assembly was unsuccessful as the proportion of viral reads was lower. A MIRA reference guided assembly using the completed SpaA1 sequence suggested the phage were highly similar, and PCR primers were designed to close the gaps with additional Sanger capillary sequencing to confirm this. The final BceA1 assembly was completed manually. Sequences of the viruses have been submitted to the EMBL European Nucleotide Archive with accession numbers HE614281 (SpaA1) and HE614282 (BceA1).

### Cohesive Ends

To determine the sequences of the SpaA1 genome termini, PCR with primers annealing close to and directed towards genome ends was performed using SpaA1 DNA as a template. The appearance of a distinct PCR product was observed. Sequence analysis of the PCR product and the SpeA1 genome end sequences determined by primer walking revealed that the PCR product contained nine extra base-pairs at the junction site between the viral DNA ends. The presence of these extra base-pairs indicates that the ends of the SpeA1 genome form cohesive 3′ overhangs.

### Annotation and Comparison of the Genomes and Phylogenetic Tree Reconstruction

An initial set of gene predictions was generated using GeneMark.hmm [Version 2.8] [32]. These predictions were then refined and annotated manually using results of searches against the non-redundant protein sequence database (NCBI, NIH, Bethesda) using PSI-BLAST [33] and the Conserved Domain Database using RPS-BLAST [34]. For each ORF within the OFR1-ORF18 region, up to 200 best PSI-BLAST hits were collected and the taxonomic distribution of the best hits was generated. The MUSCLE program [58] was used for construction of multiple amino acid sequence alignments. Maximum likelihood (ML) phylogenetic trees were constructed using the MOLPHY program [59] with the JTT substitution matrix to perform local rearrangement of an original Fitch tree [60]. MOLPHY was used also to calculate bootstrap probability which was estimated for each internal branch by using the resampling of estimated log-likelihoods (RELL) method with 10,000 bootstrap replications. Figure 2 was drawn using GenomeDiagram [61] and Biopython [62].
